# Human–animal chimeras: ethical issues about farming chimeric animals bearing human organs

**DOI:** 10.1186/s13287-016-0345-9

**Published:** 2016-06-29

**Authors:** Rodolphe Bourret, Eric Martinez, François Vialla, Chloé Giquel, Aurélie Thonnat-Marin, John De Vos

**Affiliations:** CHU Montpellier, Innovation and Research Division, Montpellier, F34000 France; Université de Montpellier, UFR de Droit, Montpellier, F34000 France; INSERM, U1183, Montpellier, F34000 France; Université de Montpellier, UFR de Médecine, Montpellier, F34000 France; CHU Montpellier, Department of Cell and Tissue Engineering, Hospital Saint-Eloi, 80 Avenue Augustin Fliche, 34295 Montpellier, Cedex 5 France

**Keywords:** Human organs, Animals, Chimera, Interspecies chimera, Animals Containing Human Material, Induced pluripotent stem cells

## Abstract

Recent advances in stem cells and gene engineering have paved the way for the generation of interspecies chimeras, such as animals bearing an organ from another species. The production of a rat pancreas by a mouse has demonstrated the feasibility of this approach. The next step will be the generation of larger chimeric animals, such as pigs bearing human organs. Because of the dramatic organ shortage for transplantation, the medical needs for such a transgressive practice are indisputable. However, there are serious technical barriers and complex ethical issues that must be discussed and solved before producing human organs in animals. The main ethical issues are the risks of consciousness and of human features in the chimeric animal due to a too high contribution of human cells to the brain, in the first case, or for instance to limbs, in the second. Another critical point concerns the production of human gametes by such chimeric animals. These worst-case scenarios are obviously unacceptable and must be strictly monitored by careful risk assessment, and, if necessary, technically prevented. The public must be associated with this ethical debate. Scientists and physicians have a critical role in explaining the medical needs, the advantages and limits of this potential medical procedure, and the ethical boundaries that must not be trespassed. If these prerequisites are met, acceptance of such a new, borderline medical procedure may prevail, as happened before for in-vitro fertilization or preimplantation genetic diagnosis.

## Background

The idea of chimeras can be traced back to Antiquity. In Greek mythology the Minotaur had a man’s body and a bull’s head, and Pan was half man, half goat. Similarly, many Egyptian gods had a human body and a beast head, such as Sobek, Anubis, and Horus. The concept of “chimera” has gone through a semantic shift since Antiquity. Originally, “Chimera” was a proper noun designating a fabulous creature, whereas in modern medicine “chimera” describes a living organism that contains cells or tissues with different genotypes. Nevertheless, there are variations to the exact meaning of this word, depending on the field. In embryology, “chimera” refers to a combination of cells from different individuals. In molecular genetics, “chimera” describes the combination of two DNA molecules from different individuals, or from different chromosomes of the same individual. Conversely, in genetics, “chimera” refers to interspecies hybrids, such as the mule (the cross of a female horse with a male donkey) [1]. “Chimera” may even refer to the grafting in a postimplantation embryo of cells or tissues from another individual or species, such as the injection of hematopoietic stem cells intraperitoneally into a sheep fetus to produce a chimeric sheep that expresses human myeloid and lymphoid lineages [2]. In the rest of this article, “chimera” will refer to the meaning used in embryology.

One of the first embryological chimeras created by scientists was the result of landmark experiments carried out by Hans Spemann and Hilde Mangold, who grafted part of one amphibian (Triturus) embryo into another with a different degree of pigmentation [3]. Later, Nicole Le Douarin et al. [3] used chimeric embryos from chicken and quails for cell lineage tracking analyses during early vertebrate development. Alongside these man-made chimeras, natural chimeras have also been described. For instance, mothers might retain some of their fetus cells after pregnancy, a phenomenon called fetal microchimerism [4].

Recent technological progress (described in the following) accomplished in the field of chimera research could now allow the production of human organs in animals and thus the generation of human–animal chimeras. The medical needs are undeniable, particularly for organ transplantation, due to the severe organ shortage [5]. Nevertheless, such a perspective raises major legal and ethical questions. This review will describe briefly the technology that allows the creation of chimeric animals bearing human organs. The review will then discuss the ethical issues raised by this possibility.

## Chimeric animals bearing human organs

### Pluripotent cells

The idea of producing human organs in animals originates from the discovery of pluripotent stem cells (PSC). Such cells can differentiate into any cell types of the organism, for instance skin, liver, or heart cells. Pluripotency is a key property of very specific human embryonic cells found in the inner cell mass (ICM) of the early embryo and that can be derived in vitro into lines of embryonic stem cells (ESC) [6, 7]. As human ESC retain the pluripotency property of ICM cells, they are particularly interesting for studying human embryo development in vitro and for regenerative medicine. Nevertheless, the origin of human ESC has raised important ethical questions because their production involves the destruction of human embryos [8].

Another source of PSC are induced pluripotent stem cells (iPSC) that result from reprogramming differentiated cells into pluripotent cells by transitionally forcing the expression of four transcription factors [9, 10]. These iPSC have the same properties as ESC. The possibility to produce pluripotent cells from adult cells and not from ICM cells has many medical and scientific applications. For example, it would be possible to produce autologous medicinal cells for regenerative medicine, or to derive iPSC from patients with a genetic disease to model the disease in Petri dishes. In 2012 Shinya Yamanaka, who invented induced pluripotent stem technology, was awarded the Nobel Prize in Medicine for this discovery.

### Chimeric animals by blastocyst complementation

Remarkably, mouse ESC injected into a blastocyst can colonize its ICM and contribute to embryo development [11]. If the blastocyst and ESC have a different genetic background (e.g., blastocyst of a white mouse and ESC of a black mouse) the outcome is a bicolored mouse and its organs will include cells of both origins. These mice are called “chimeras”. This technology quickly became a way to produce transgenic mice. Indeed, if ESC are genetically modified before their introduction into the blastocysts, these genetic modifications will be transmitted to part of the chimeric mouse cells, including the gametes. The inventors of this technology were awarded the Nobel Prize in Medicine in 2007 [12].

### Interspecies chimeric animals and production of xenogeneic organs

In 2010, the team of Hiromitsu Nakauchi reported that injection of rat iPSC into mouse blastocysts produced mice with organs that also included rat cells [13]. Similar results were obtained when mouse iPSC were injected into rat blastocysts. Surprisingly, when rat iPSC were injected into mouse blastocysts in which the *Pdx1* gene that is essential for pancreas development was invalidated, newborn chimeric mice bore a rat pancreas and had mostly a normal life [13]. Conversely, *Pdx1*^–/–^ mice are born without a pancreas and quickly die after birth due to lack of insulin. Rat iPSC thus took the place of the deficient mouse cells that could not contribute to pancreas development (i.e., blastocyst complementation). The same approach could be exploited to produce many other xenogeneic organs in mice [14].

### Production of human organs in animals

The demonstration that a rat organ can be produced in a mouse suggests it could be possible to produce xenogeneic organs (including human organs) in various animal species. For production of human organs, the carrier animal would be altered genetically in order to block the development of a given organ. Human iPSC would then be injected into blastocysts obtained from such genetically altered animals. Thus, only human cells would contribute to the development of that organ [15]. Importantly, by using autologous iPSC it might be possible to produce autologous human organs and as a consequence reduce the long-term need for immunosuppressive drugs after transplantation. These chimeric animals would be raised until the human organ reached the required size and then they would be sacrificed on transplantation day.

Even if alternative animal choices may be considered, pigs make attractive animals to bear human organs for various reasons. First, the size of their organs is similar to the size of human organs. Also, their metabolism (for instance, diet and temperature) is close to the human metabolism. Finally, there is an important corpus of knowledge about pig cell administration in humans within the context of xenotransplantation trials. This knowledge will facilitate the identification of hazards and barriers, such as infectious and immunological risks, to be overcome for transplantation in humans of human cells or organs that have been grown in a pig.

Some preliminary experiments have already been performed, such as injection of human ESC into a mouse blastocyst [16]. This first experiment failed; however, more recent attempts using human iPSC led to the production of mice with a significant percentage of chimerism. These mouse embryos were sacrificed at early developmental stages for ethical reasons [17]. Injection of macaque rhesus ESC into mice blastocysts also led to a significant proportion of chimerism [18].

Many technical barriers have to be overcome before the production of human organs in pigs becomes a reality. In addition, some safety concerns must be addressed, such as the risk of zoonosis (see later) and the contamination of human organs by residual animal cells or animal proteins that could elicit potentially deleterious immune reactions in the organ receivers [15]. Nevertheless, in view of the major medical/economic issues linked to this new technology, much research is currently focused on these issues [14, 19, 20].

### Ethical issues

The possibility to create animal chimeras carrying human organs is associated with many and important ethical objections. In our view, these legitimate ethical concerns must be analyzed rigorously and addressed with appropriate technical handling, as discussed in the next sections. Ultimately, properly discussing such ethical issues and finding a general consensus will pave the way for the development and acceptance of this new technique in the patients’ interest.

As a preliminary remark, it must be noted that, to a certain extent, these ethical considerations were raised previously by medical techniques that today are widely accepted and that also allowed the creation of human–animal chimeras. For instance, porcine, bovine, and equine biological heart valves are often implanted in patients with cardiac valve dysfunction and insulin extracted from porcine pancreas is routinely used by patients with diabetes. Is it justified to consider that a pig carrying a pancreas of human origin is a chimera the production of which should be forbidden, whereas a human being with a mitral valve of porcine origin is a permissible chimera?

To this question, two answers are most likely to be provided, depending on the adopted perspective. Concerning the animal welfare, experimentations on pigs are already strictly regulated to avoid unnecessary animal suffering during research. The fact that chimeric animals are raised for the purpose of human organ culture should not face more ethical debates than raising them for consumption.

Concerning the production of chimeras, some ethical issues are closely tied to medical concerns. Indeed, one main worry is that the retroviruses integrated in the genome of animals could be transferred to humans. Indeed, the effects of these retroviruses might be known in animals, but there is no possibility of predicting what they could cause in humans. The fear is that human tissues produced in animals might be the source of new zoonoses, which brings up the ethical problems linked to the protection of human participants in clinical research to test the safety of such organs [21, 22]. Moreover, the impossibility to anticipate the potential risks associated with the transplantation of human organs grown in pigs calls for caution.

In addition, crossing the species boundaries between humans and animals is a major ethical concern and could represent a source of resistance by the general public [23, 24]. Although the idea of mixing human and animal genes or cells is not often considered by the general public and the notion of chimera does not necessarily convey the scientific reality to the general public, it is already a standard practice in science [25]. The created entities are covered by the term “Animals Containing Human Material” (ACHM) [26, 27]. For instance, mice have been genetically modified to make them more susceptible to infection by human viruses, such as HIV [28]. Moreover, human neuronal cells have been injected into the brain of mice [29]. Similarly, transgenic animals can produce human proteins in their milk, such as human anti-thrombin in goat milk for the treatment of blood-clotting disorders [30]. To respond to the public concerns about the unnaturalness stemming from human–pig chimeras, it will be important to openly discuss the key ethical issues (see later), but also, as underlined by Nakauchi, to explain the medical reasons for the project (i.e., to treat desperately ill people in the absence of suitable alternatives) [31].

The production of human organs in animals also raises legal issues and is subject to different legal frameworks in different countries (Table 1). These differences will most likely affect the speed of development and implementation of this organ production strategy in different countries. For instance, when Nakauchi wished to move from research on rat pancreas production in mice to human pancreas generation in pigs, he had to relocate his research group to Stanford (USA) from Kyoto (Japan) because of Japanese opposition to such research. Nevertheless, he also faced difficulties in obtaining federal financial support in the USA [32, 33].

**Table 1:** Research policies on human-animal chimeric embryos and chimeric animals

Country	Policies and recommendations
France	French law stipulates that creating a chimeric human embryo is forbidden. The law clearly forbids the introduction of allogeneic or xenogeneic cells into a human embryo; however, it is unclear whether it also bans the introduction of human cells into animal embryos. We discussed this uncertainty elsewhere [47].
UK	The Human Fertilization and Embryology Act 1990, as amended by the Human Fertilization and Embryology Act 2008, applies to embryos that are either entirely or predominantly human or equally human and animal. It does not forbid the creation of animal chimeras by grafting human embryonic cells or embryonic cell lines into animals. However, transferring a human admixed embryo, which would be predominantly human, into an animal foster “mother” is forbidden. Of note, the definition of “predominantly human” is a crucial point, although admittedly difficult to resolve (http://www.acmedsci.ac.uk/download.php?f=file&i=13666).
Germany	The German law forbids combining a human embryo with animal cells, but not the introduction of human cells into an animal embryo. The German Ethics Council (Deutscher Ethikrat) published an opinion paper in September 2011 on the use of human–animal mixtures in research [48]. This paper highlighted the importance of finding a balance between expected medical benefits, respect of the animal welfare, and, overall, the need for an interdisciplinary scientific discussion on this subject (www.ethikrat.org/files/opinion-human-animal-mixtures-in-research.pdf).
USA	Federal US laws do not rule this issue. Nonetheless, in 2005, “the U.S. National Research Council and the Institute of Medicine recommended limits on such research, among them that no human stem cells be added to primate embryos and that animal-human chimeras not be allowed to breed” [32].
Japan	The Japanese law currently limits research on human–animal chimeric embryos by not allowing the development of such embryos beyond the appearance of the primitive streak (maximum 14 days post fertilization) and their transfer into an animal uterus. Recently, the Japanese Expert Panel on Bioethics [49] supported the idea of creating a human––animal chimera and proposed that the Japanese research regulation “should be capable of responding flexibly to the advances in research”.

In the following, we discuss the major ethical issues concerning the creation of animal chimeras. They mainly stem from the fear of species-boundary crossing, namely the risk of development of human-like consciousness in chimeric animals, or the risk of the appearance of human-like external features, and, finally, the risk of human gamete production.

### Human-like consciousness

It is our brain that makes us humans [26]. Therefore, it is crucial to determine whether injection of human iPSC into animal blastocysts could humanize the animal brain. Specifically, could a significant contribution of human cells to the animal brain modify the characteristics of the recipient species [34]? Would this affect the evaluation of the moral status of the animal, especially in the case of large animals, such as pigs and particularly nonhuman primates? Indeed, if the presence of human cells in the animal brain resulted in a form of human-like consciousness, such scientific experiments would become ethically unacceptable because a major distinction between humans and animals is based on consciousness. Surely, if chimeric animals were to acquire a human-like consciousness, it would be necessary to treat them as humans and consequently any form of organ culture would be prohibited [35]. However, this is very unlikely. As discussed by Karpowicz et al. [1], neural progenitor cells need more time to develop and go through many more divisions and result in a larger brain in primates than in most other animals. Hence, the formation of a large human-like brain within the time and space limits imposed by a nonhuman host is highly improbable. For example, the sow’s gestation period is approximately 3 months, one-third of a human pregnancy and far shorter than that of extremely preterm babies. Therefore, even if human neural progenitors would take over, the timeframe of a saw’s gestation would not be long enough to allow the full development of the neural structures needed to give rise to human functions and behaviors.

Nevertheless, there are several ways to limit this risk. First, iPSC should be injected only for complementation. In this setting, human cells would mostly replace cells/tissues that are absent as a consequence of gene knockout, as done in the *Pdx1*^–/–^ mice. Human cells are expected to be less competitive than pig cells in a pig microenvironment, unless a specific pig cell type has been impaired by genetic means. This considerably limits the risk of significant animal brain colonization by human cells. Nevertheless, certain human cell types may have an intrinsic competitive advantage over animal cells, particularly in the brain, and this can raise concerns. This was shown recently by injecting human glial progenitor cells into immunodeficient neonate mice. Human glial progenitor cells outcompeted mouse glial progenitor cells and ultimately, in these mice, the white matter was largely of human origin [36]. Strikingly, engraftment of human glial progenitor cells enhances synaptic plasticity and learning in adult mice [29].

To prevent the worst-case scenario of a humanized pig brain, it will be mandatory to define a maximal limit of human chimerism in the pig brain that cannot be exceeded. To this aim, brain chimerism could be assessed in a significant number of chimeric animals sacrificed at different time points during gestation and then after birth at different ages. Moreover, the injected human iPSC could be genetically modified to render them incapable of differentiating into neural cells, or could include “suicide genes” that would only be activated upon neural differentiation [15]. Finally, a most recent proposition is the forced expression of the *MIXL*1 gene that forces cells into a visceral fate, thus avoiding the danger of neural differentiation [20].

### Human-like external features

It may be argued that the injection of human iPSC into animal embryos could have an effect on the physical aspect of the animal; in other words, on its appearance. The creation of human/animal chimeras can make the boundary between human beings and other living beings porous, inducing questions about our human identity. These interrogations and concerns are more obvious when it comes to a chimera whose physical attributes would let its chimeric quality explicitly appear. It must be said that our moral intuition is particularly influenced by identity attributes. Thus, in a report related to human/animal chimeras, the German Ethics Committee considered that “aspects of the visible form of a living organism that are considered, in particular, to be relevant to identity, may strongly influence their intuitive ontological classification” [37]. It is not only about the creature’s appearance, but also about its specific attributes, such as language.

Previous studies concluded that the donor cell contribution is almost 20 % in rat–mouse chimeras. This is less important than in allogeneic (mouse–mouse) chimeras where it is estimated at 50 %. On the basis of these observations, researchers hypothesize that the contribution of human cells injected into pig blastocysts should be lower than 1 % [15]. Therefore, the risk of seeing the birth of pigs with human features is expected to be very low [38]. Here again, a maximum threshold of human cell contribution should be established beforehand and strictly implemented. This risk could be further limited by performing a prebirth systematic diagnosis to detect the presence of human features. If, for instance, a pig embryo had a hand or a foot, it would be sacrificed. Ultimately, only fetuses without any human feature would be allowed to term.

Overall, the risk of seeing human features in a chimeric animal seems limited and controllable.

### Human gamete production

Humanization of animals bearing human organs could also result in the production of human gametes. Human embryos could thus be created using such gametes. This would make their filiation the most “tortuous, having passed by the intermediation of a chimera” [39]. The worst-case scenario would be that a pig producing human sperm could accidently mate with a sow or vice versa. However, the possibility that the interaction between gametes of different species would result in a hybrid embryo is almost nonexistent, because the interspecies reproductive barrier is very strong. For instance, the injection of human sperm into a hamster egg (the “hamster test”, used to test the quality of human sperm cells) does not give rise to embryos capable of development [40]. Even cross-breeding attempts between human and anthropoid apes failed when tested in the first part of the twentieth century [39]. In addition, this fear can easily be dissipated: sterilization of pigs bearing human organs would be sufficient to prevent their reproduction. Alternatively, the injected human iPSC could be genetically modified, similarly to that proposed to prevent human brain development, to inhibit their differentiation into gametes, or could include “suicide genes” that would only be activated upon germinal differentiation.

### Alternatives to human organs in animals

As discussed earlier, the absence of medical alternatives is essential to justify the development of human organs in animals. However, the issue of organ shortage for the treatment of life-threatening conditions by organ transplantation could be solved by other means in the future. For instance, pig organs could be used for xenotransplantation. To prevent organ rejection or zoonosis transmission, researchers are investigating the possibility of deleting the main pig genes responsible for xenogeneic organ rejection [41] and of breeding pigs in which all porcine endogenous retroviruses are inactivated using CRISPR/Cas9 technology [42]. Alternatively, human PSC may be differentiated in vitro for cell-based therapies of various diseases [43, 44]. A recent advance in this field was the exploitation of PSC self-organizing properties in vitro to form three-dimensional tissues, called “organoids”, with therapeutic potential, including for the treatment of diabetes [45]. It is now important to monitor their development because they might represent valid and more acceptable alternatives to the generation of interspecies chimeras.

## Conclusion

If the production of animal chimeras carrying human organs is facing several obstacles, none of the ethical issues seems insurmountable. Indeed, an important humanization of such animals is not expected in view of the overall modest contribution of human cells and of the availability of techniques to target a specific organ [15]. As stated earlier, establishing a threshold of chimerism will be mandatory to set acceptable limits for the animals’ humanization and for the humans’ animalization.

In any event, the therapeutic benefits of such an approach would be immense. Only in Europe, more than 60,000 people were on the organ transplant waiting list in 2013 [46]. Growing human organs in pigs would allow addressing the chronic organ shortage and establishing a stock of available organs to avoid placing people on the waiting list for a suitable donor. Currently, this strategy focuses on the pancreas, but could be extended to other organs, such as the kidneys [14], and could lead to the eradication of organ shortage.

Fear born from ignorance will naturally result in a spontaneous reluctance towards or rejection of the concept of animals bearing human organs. These ethical issues must therefore be debated now and the public involved in these discussions. Scientists and physicians must explain the medical needs, carefully describe the techniques used, and indicate the ethical limits that cannot be trespassed.

## Abbreviations

ACHM, Animals Containing Human Material; ESC, embryonic stem cells; ICM, inner cell mass; iPSC, induced pluripotent stem cells
